# Marsupialization of a large dentigerous cyst in the mandible with orthodontic extrusion of three impacted teeth. A case report

**DOI:** 10.4317/jced.53890

**Published:** 2017-09-01

**Authors:** Nedal Abu-Mostafa, Arshad Abbasi

**Affiliations:** 1 Assistant Professor in Oral and Maxillofacial Surgery and Diagnostic Science Department, Riyadh Colleges of Dentistry and Pharmacy, Kingdom of Saudi Arabia; 2 Lecturer in Orthodontics, Preventive Dentistry Department, Riyadh Colleges of Dentistry and Pharmacy, Kingdom of Saudi Arabia

## Abstract

The dentigerous cyst (DC) is the most common type of developmental odontogenic cyst. It is usually asymptomatic and associated with the crown of an unerupted or impacted tooth. However, after a long duration, it is likely to cause significant bone resorption, cortical expansion, and tooth displacement. This report presents a large infected DC in the mandible of a 12-year-old female patient. The DC was located inferior to badly decayed primary molars and surrounded three impacted permanent teeth: canine, first premolar, which had a dilacerated root, and second premolar. The DC was treated successfully by marsupialization and extrusion of the impacted teeth. In conclusion, the combination of marsupialization with orthodontic extrusion is a conservative, efficient protocol that stimulates bone healing and promotes the eruption of cyst-associated teeth even if they are deeply impacted, crowded, or have a dilacerated root.

** Key words:**Dentigerous cyst, marsupialization, impacted teeth, orthodontic extrusion, dilacerated root.

## Introduction

The dentigerous cyst is defined as an epithelial-lined pathologic space associated with the crown of an unerupted or impacted tooth. It most frequently involves third molars, maxillary canines, and premolars, but it may also involve unerupted supernumerary teeth or odontomas (1).

The dentigerous cyst results from an accumulation of fluid between the reduced enamel epithelium and the enamel or between the layers of the enamel organ. The lining of a dentigerous cyst is non-keratinized stratified squamous epithelium (2). On the other hand, the epithelial cells are capable of producing metaplastic changes and progressing to more aggressive lesions such as odontogenic keratocysts, ameloblastoma, mucoepidermoid carcinoma, or squamous cell carcinoma (3).

This cyst is usually asymptomatic and discovered incidentally by routine radiographic examination; however, pain may occur when it become infected. After a long duration, it is likely to cause significant bone resorption, cortical expansion, and teeth displacement, particularly in the cyst-associated tooth. The radiographic appearance of a dentigerous cyst is well-circumscribed unilocular pericoronal radiolucency (more than 5 mm) with an unerupted tooth (4).

Treatment of a dentigerous cyst includes enucleation or marsupialization. Enucleation is the modality of treatment that includes complete removal of the cystic lining and extraction of the impacted tooth. This type of treatment is indicated when the cyst surrounds a supernumerary tooth or if the cyst-associated tooth is not expected to erupt either spontaneously or by extrusion (5).

Marsupialization is a conservative surgical intervention that decreases the size of the cyst gradually. The procedure involves making a window on the cystic wall by incision, evacuation of the contents of the cyst, and suturing the cystic lining to the oral mucosa. It has advantages in promoting eruption of the cyst-associated tooth with or without orthodontic traction. On the other hand, the disadvantages of marsupialization include the long duration of treatment and leaving the larger part of the cystic lining in situ (6).

This report presents a large infected dentigerous cyst in the mandible of a young female patient. The cyst surrounds three impacted teeth: canine, first premolar, and second premolar. It was treated successfully by marsupialization and orthodontic extrusion of the impacted teeth.

## Case Report

A 12-year-old medically fit female patient presented to the College’s clinics on April 19, 2015, with swelling on the left side of the lower jaw. The patient had a history of facial swelling which started three months ago; however, the swelling had become painful and increased in size rapidly within the last two weeks. The patient visited a general dentist who ordered a panoramic radiograph which showed a large lesion on the bone of the lower jaw associated with three impeded teeth. The dentist prescribed antibiotics and asked her to visit an oral and maxillofacial surgeon. Then the swelling regressed in size within a few days. This case report was registered in the College’s Research Center with registration number FRP/2016/96. The FDI World Dental Federation notation was used for the numbering of teeth in this report.

-Clinical examination

Extra-orally, the face was asymmetric with a small swelling on the left side of the body of the mandible. The overlying skin was normal and submandibular lymph nodes were not palpable. No alteration in sensation was noticed in the lower lip. Intra-orally, there were poor restorations on 74 and 75 with recurrent caries. Hard bony swelling was found on the left body of the mandible extending from the mesial of 73 to the distal of 75. The overlying mucosa was normal and freely movable.

**Figure 1:**
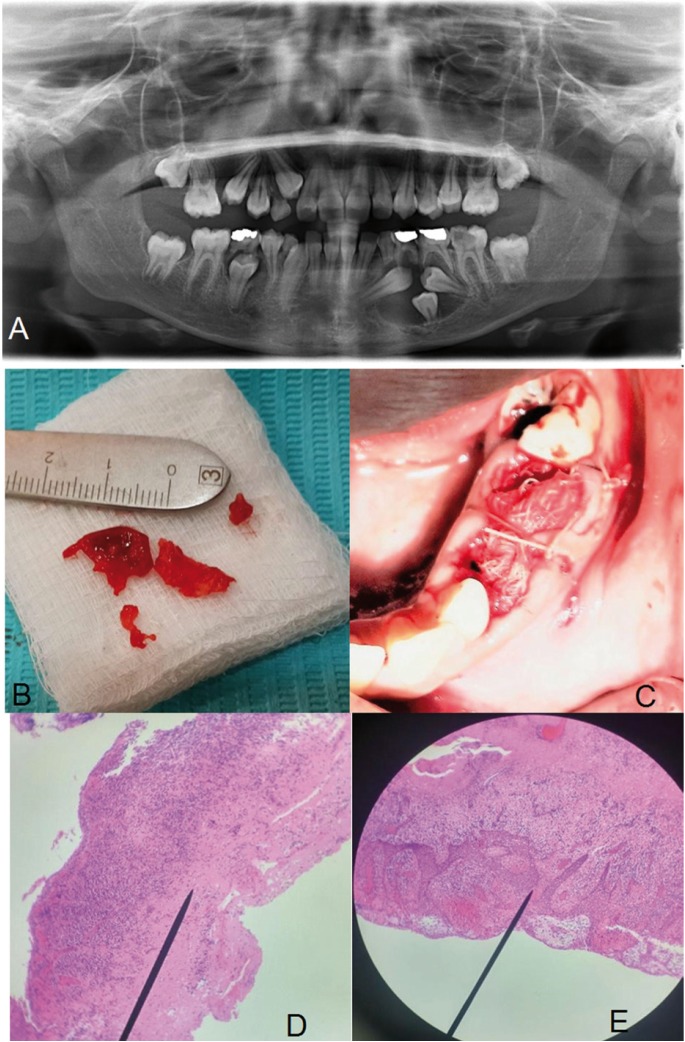
A) Preoperative panoramic radiograph showed a large dentigerous cyst associated with impacted 33, 34, and 35. B) The excised roof of cystic lining and bone were taken as biopsy. (C) Sutures were placed on the papilla and across the pack of gauze. (D) A histopathologic view shows inflamed connective tissue capsule (hematoxylin–eosin staining; original magnification X40). (E) A histopathologic view shows degenerative epithelium and inflammatory cells (hematoxylin–eosin staining; original magnification X100).

Radiographic examination included a panoramic radiograph (Fig. 1A) and periapical films which revealed a large radiolucent lesion with a well-defined radiopaque margin on the left side of the mandible. The lesion extended from the distal surface of 32 to the mesial surface of 36. It surrounded impacted 33, 34, and 35 which had incomplete roots and open apices. Impacted 33 had distal angulation, while 34 was vertically impacted with a dilacerated root. Impacted 35 had mesial angulation.

Other findings on the left side of the mandible included retained 73 with root resorption and deep mesial caries on 36. Findings on the right side of the mandible comprised retained 83, retained 85 with amalgam restoration, and unerupted 45. On the right side of the maxilla, the findings were retained 53, unerupted 13, and incompletely erupted 15.

Orthodontic examination showed a convex facial profile with average facial height and competent lips. Generalized spacing was noticed in the upper arch with rotation on 14 and 24. Upper and lower second molars were unerupted bilaterally. A slightly in-creased overjet and overbite were noticed.

The differential diagnosis of the lesion was a dentigerous cyst with infection from the primary molars. The authors’ plan was to extract 74 and 75 followed by marsupialization of the cyst and incisional biopsy. The next step included the alignment of teeth and overbite correction, then forced extrusion of the impacted teeth.

-Surgical operation

Preoperatively, the patient rinsed with 2% chlorohexidine mouthwash. Local anesthesia was achieved by inferior alveolar nerve block 2% lidocaine with 1:80,000 epinephrine. Also, buccal mucosa was anesthetized by infiltration close to the first molar. Diagnostic aspiration was done by syringe size 5 cc and showed whitish fluid mixed with blood. Primary 74 and 75 were simply extracted without disruption of the cystic lining. A muco-periosteal flap was reflected by sulcular and distal releasing incisions. The cystic lining was unroofed by an incision at the site of the extracted primary teeth, then a part of the inter-septal bone was removed (Fig. 1B). The cyst fluid was evacuated, followed by copious irrigation with normal saline. The impacted teeth were inspected and the margin of the cystic lining was sutured to the adjacent gingival margin of the wound. A pack of gauze soaked with Fusidic acid (Fucidin cream 2%) was applied inside the cavity up to the extraction site. The releasing incision was sutured by 4-0 polyglycolic acid (PGA RESORBA, RESORBA Medical GmbH, Nurnberg, Germany). Other sutures were placed on the papilla distal to the canine, the papilla mesial to the first molar, and across the gauze to stabilize it (Fig. 1C). Postoperative medications included Amoxicillin/ Clavulanic Acid (Augmentin, GlaxoSmithKline) 625 mg P.O. every 8 hours for five days, Ibuprofen (Brufen, Hamol Limited, Nottingham, England) 400 mg P.O. every 8 hours for three days, and 2% Chlorohexidine mouthwash every 12 hours for seven days. The excised lining, bone, and aspirated fluid were sent to the pathology laboratory for histopathologic examination.

The patient came on the third and seventh day after the operation for follow-up. No change in sensation in the lower lip was noticed. The wound was irrigated with normal saline and oral hygiene instructions were given.

Fourteen days post-operatively, soft tissue healing was uneventful. The sutures and the pack of gauze were removed gently, and no pus discharge was noticed. The cavity was irrigated with normal saline then a new smaller pack of gauze soaked with Fucidin cream was applied. Packing had been replaced regularly every seven days. The biopsy result confirmed the differential diagnosis, which was infected dentigerous cyst (Fig. 1D,E). Five weeks post-operatively, the pack of gauze was removed (Fig. 1A) and 83 and 85 were extracted. The patient was instructed to irrigate the cystic space with normal saline at least two times daily and she was recalled periodically for follow-up. Root canal treatment was performed for 36 in a private dental clinic.

**Figure 2:**
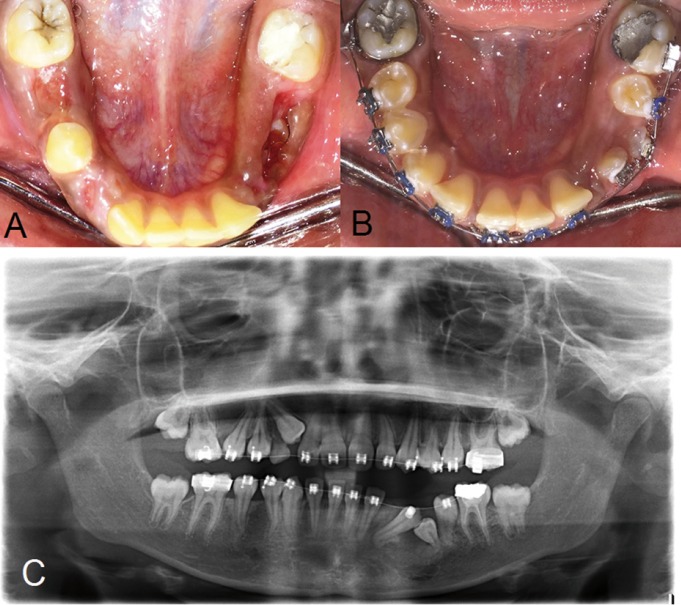
A) Soft tissue healing after removal of the pack of gauze in the 5th week post-operatively. B,C) Clinical picture and panoramic radiograph after five months of orthodontic treatment which revealed the up-righting of 35 and slight spontaneous eruption of 34.

-Orthodontic treatment

After three months, a panoramic radiograph showed a decrease in the radiolucency at the site of the cystic cavity. Orthodontic treatment started by bonding a 0.022-inch x 0.028-inch slot pre-adjusted appliance to the available teeth. Then light continuous arch wires were placed to allow the leveling of teeth and overbite correction simultaneously. The initial wire was 014-inch Nickel Titanium for the upper and lower arches.

After the leveling of teeth, the next step was up-righting distally impacted 33 and mesially impacted 35. A 16-inch x 22-inch stainless steel (SS) wire was stabilized on the lower arch. The impacted teeth were re-exposed by reflection of a small buccal flap. Then a power (E) chain was engaged from 43 to 33 and from 35 to 36 to up-right them, which later provided space for the guided eruption of deeply embedded 34. After five months of orthodontic treatment, clinical examination and panoramic radiograph revealed the up-righting of 35 and slight spontaneous eruption of 34 (Fig. 2 B,C). On the other hand, a substandard root canal treatment was noticed in 36, which later was redone by an endodontist in the College. After up-righting of 33, 34 was bonded and aligned. In the upper arch, retained 53 was extracted and then impacted 13 was surgically exposed by an apically positioned flap. Orthodontic traction for this canine was performed using two wires simultaneously. A 0.016-inch x 0.022-inch SS stabilizing base wire was ligated then a 0.014-inch NiTi was ligated as an active wire over it to engage 13. The idea was to prevent the inward tipping of the adjacent lateral incisor and first premolar during orthodontic forced eruption of the canine.

The patient was recalled monthly for reevaluation and to make the required adjustments for the appliance.

**Figure 3:**
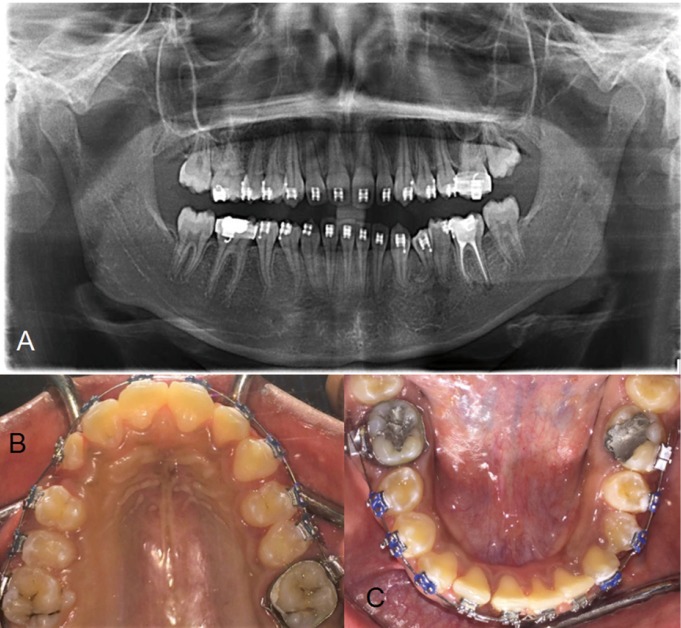
A) Panoramic radiograph after alignment of teeth and healing of the cystic cavity. B) Eruption of teeth in the upper arch which still need slight alignment. C) Uneventful eruption, and alignment of the impacted teeth in the lower arch.

The duration of orthodontic treatment was 35 months. The panoramic radiograph and clinical examination showed complete healing of the cystic cavity, uneventful eruption, and alignment of the impacted teeth (Fig. 3A,C). However, the upper arch still need slight alignment (Fig. 3B).

## Discussion

The dentigerous cysts are the most common type of developmental odontogenic cysts that develop in the second and third decades of life (2). Benn A and Altini M (7) categorized these cysts as developmental and inflammatory. The developmental type results from impaction of mature teeth and predominantly involves the mandibular third molars. It is generally discovered on routine radiographs in the late second or third decades of life. The inflammatory type involves immature permanent teeth and results from an infected necrotic primary tooth that stimulates the developing tooth germ follicle. Diagnosis of this type is established in the first and early part of the second decade. The dentigerous cyst in the present report belonged to the inflammatory type based on the patient’s age, the presence of necrotic primary molars, and the presence of pus in the lumen of the cyst. Marsupialization was the treatment of choice because enucleation in such case would sacrifice three impacted permanent teeth in an adolescent female. Tooth loss at a young age will affect the occlusion, function, and esthetic appearance. Furthermore, enucleation carries a risk of trauma to the inferior alveolar nerve and mental nerve in addition to a large postoperative bone defect.

In the current case, the roof of the cystic lining inferior to 74 and 75 was removed carefully to serve as the window on the cystic cavity. Such procedure makes the extrusion of the impacted tooth toward the normal position easy and avoids creating a window on the buccal cortex. A similar approach was used by Bozdogan E *et al.* (8) and Qian W T *et al.* (9). They preferred it to the lateral window, which can result in ectopic eruption and malocclusion of the developing permanent teeth.

Spontaneous eruption of the cyst-associated tooth is predictable and may take place three months after marsupialization; however, orthodontic extrusion may be required in some cases (10). A controversy exists about predictive indicators for the eruption of a cyst-associated tooth after marsupialization. Fujii R *et al.* (11) and Serra e Silva *et al.* (12) mentioned factors that influence tooth eruption: whether the patient is older or younger than 10 years old, tooth depth, inclination, incomplete root formation with an open apex, and sufficient space. In contrast, Yahara Y *et al.* (10) and Qian W T *et al.* (9) found that these factors are insignificant and do not affect tooth eruption.

In the present dentigerous cyst, impacted 33 was tilted distally while impacted 35 was tilted mesially and both impeded the path of eruption of 34. The crowding of impacted teeth and the dilacerated root of 34 made spontaneous eruption after marsupialization impossible. Therefore, the impacted teeth had to be extruded by orthodontic appliance. The combination of marsupialization and forced extrusion of the teeth has the advantage of decompression in the cystic cavity. Also, it creates tensional forces on the periodontal fibers, which results in the deposition of new bone along the way of tooth eruption and the residual cavity (13) 

The extrusion of an impacted tooth with a dilacerated root in normal bone is challenging for clinicians. It includes the risk of root resorption (14) or cortical bone perforation (15). Nonetheless, in this case report, 34 was extruded successfully and neither root resorption nor cortical perforation occurred. This can be explained by easier eruption of the tooth through the newly formed bone within the cystic cavity than eruption in normal bone (10). Furthermore, the mesial curvature of the dilacerated root put the apex away from the buccal and lingual cortex that contributed in the eruption without cortical perforation.

The final result of treatment was uneventful healing of the dentigerous cyst with the establishment of good occlusion. In conclusion, the combination of marsupialization with orthodontic extrusion is a conservative, efficient protocol that preserves cyst-associated teeth and promotes their eruption and bone healing. By such treatment, the eruption of impacted teeth is possible, even if they are deeply impacted, crowded, or have a dilacerated root.
